# Supplementary material for: New plastids, old proteins: repeated endosymbiotic acquisitions in kareniacean dinoflagellates
Source: EMBO Rep. 2024 Mar 18;25(4):16. doi: 10.1038/s44319-024-00103-y (PMC11014865; doi:10.1038/s44319-024-00103-y)

Appendix

New plastids, old proteins: repeated endosymbiotic acquisitions in Kareniaceae

Anna M. G. Novák Vanclová, Charlotte Nef, Zoltán Füssy, Adél Vancl, Fuhai Liu, Chris Bowler, Richard G. Dorrell

EMBO Reports, 2023

Contents:

Appendix Figure S1 2
Appendix Figure S2. 3
Appendix Figure S3. 4
Appendix Figure S4 4
Appendix Figure S5 5
Appendix Figure S6 7
Appendix Figure S7 8
Appendix Figure S8 9
Appendix Figure S9 10
Appendix Figure S10 11
Appendix Figure S11 12
Appendix Figure S12 13
Appendix Figure S13 14
Appendix Figure S14 15
Appendix Figure S15 16
Appendix Figure S16 17
Appendix Figure S17 17
Appendix Figure S18 18
Appendix Figure S19 19
Appendix Figure S20 19
Appendix Figure S21 20
Appendix Figure S22 20
Appendix Figure S23 21

Appendix Figure S1: Graphical summary of the bioinformatic pipeline used to predict plastid-targeted proteins and obtain their phylogenetic annotations.

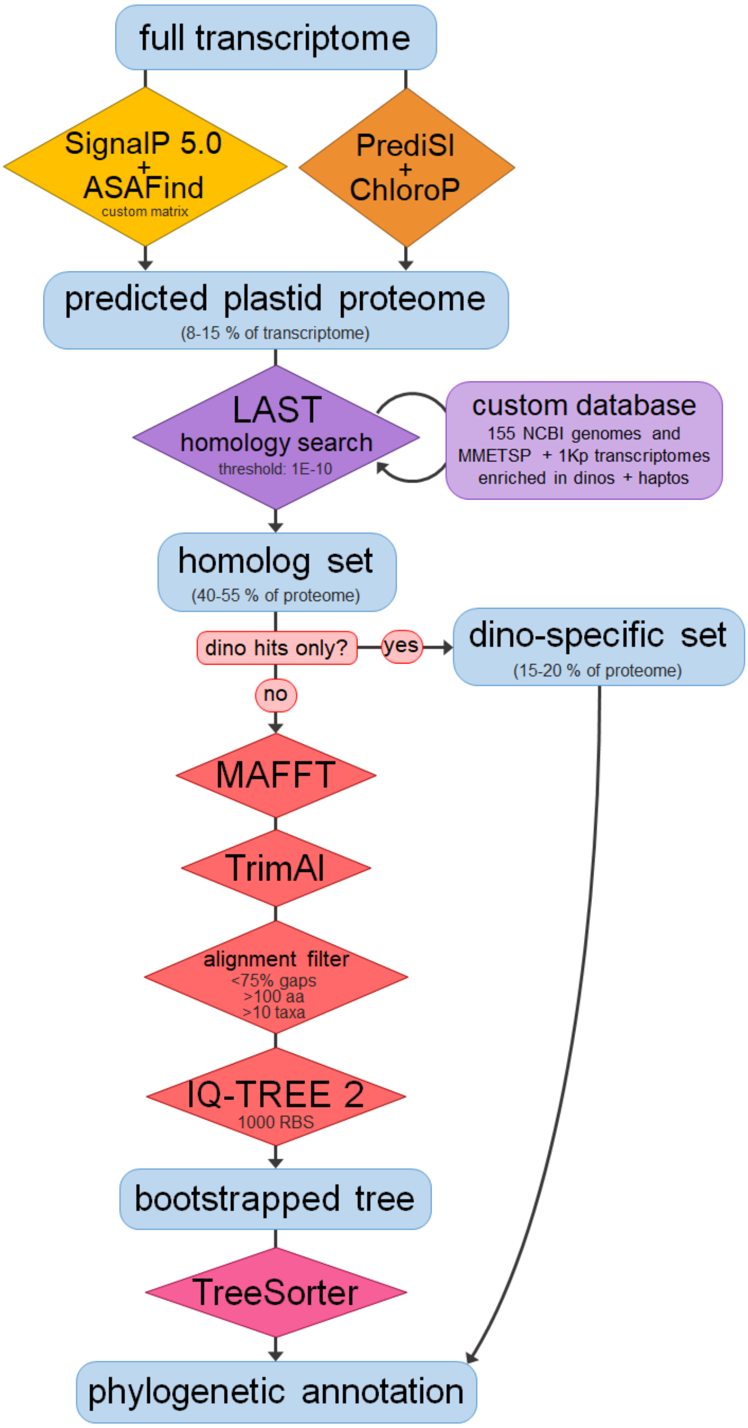

Appendix Figure S2: Proportions of single gene trees showing monophyly of two or three of the studied genera and a plastid-late origin of the gene, further sorted by phylogenetic affinity to specific haptophyte subgroups.

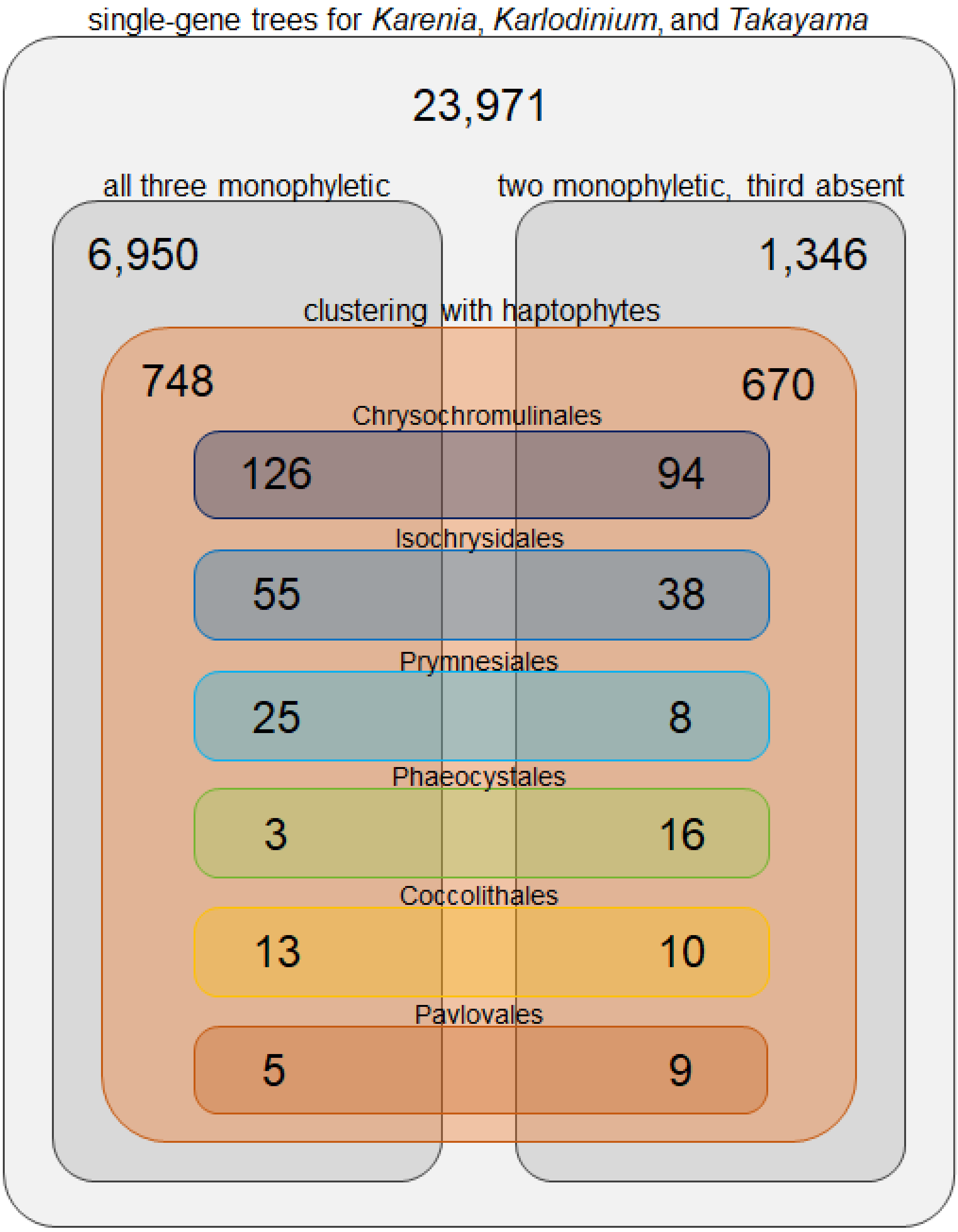

Appendix Figure S3: Ratios of the plastid-late proteins further sortable to respective haptophyte subgroups normalized against sum of numbers of sequences in all the respective haptophyte subgroup transcriptomes in our database (a), average size of these transcriptomes (b), and the maximum BUSCO score for each subgroup (c). Note the relatively small contribution of Pavlovales and Phaeocystales, especially in the case of Takayama whose current plastid was gained from the latter group.

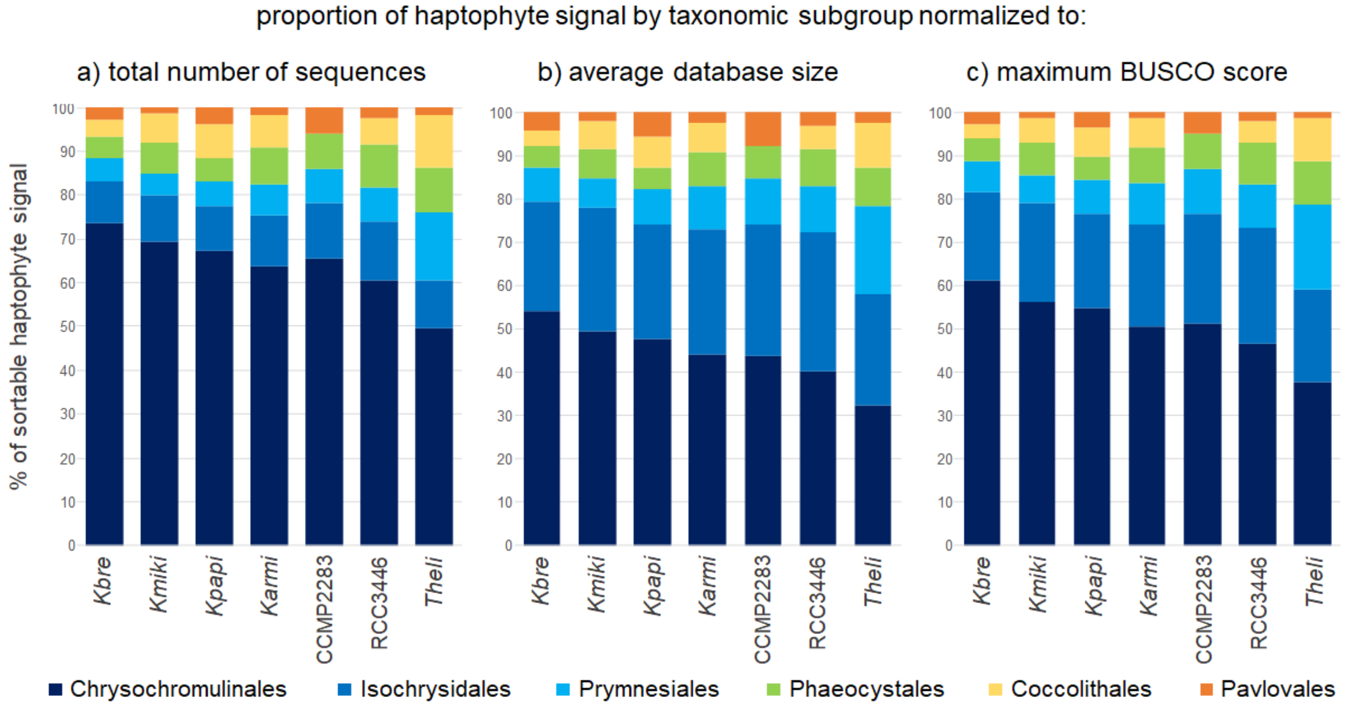

Appendix Figure S4: Venn diagram of the distribution of homologs of proteins with specific Isochrysidales-like origins recovered in fucoxanthin plastid proteomes; the number of shared homologs is the highest between Karenia and Karlodinium (41), followed by those shared by the three non-RSD genera (28), and only then those shared by all four (10). At the same time, the highest number of genus-specific Isochrysidales-like proteins are in Karenia (40) and RSD (35). In the RSD, this number is higher than the total number of proteins shared with at least one other group, reflecting the different evolutionary origin of its plastid.

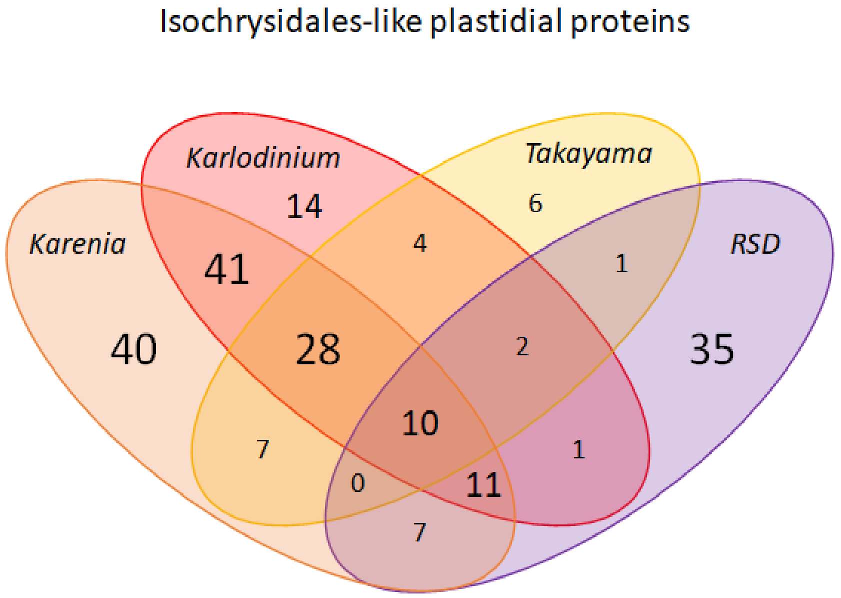

Appendix Figure S5: Single-gene tree generated by our pipeline with Karenia-brevis-SP1-CAMPEP-0189089492 (functionally annotated as acetylglutamate kinase) as a seed sequence showing the Isochrysidales-like phylogenetic affiliation of the retrieved Karenia homologs and separate, low-supported dinoflagellate-like origin of the Karlodinium homologs. A Takayama homolog was not identified for this protein while retrieved RSD homologs resolve with Phaeocystis and with bacteria. Bootstrap support is expressed by the branch colour (black for ≥90%, dark grey for ≥75%, light grey for <75%)

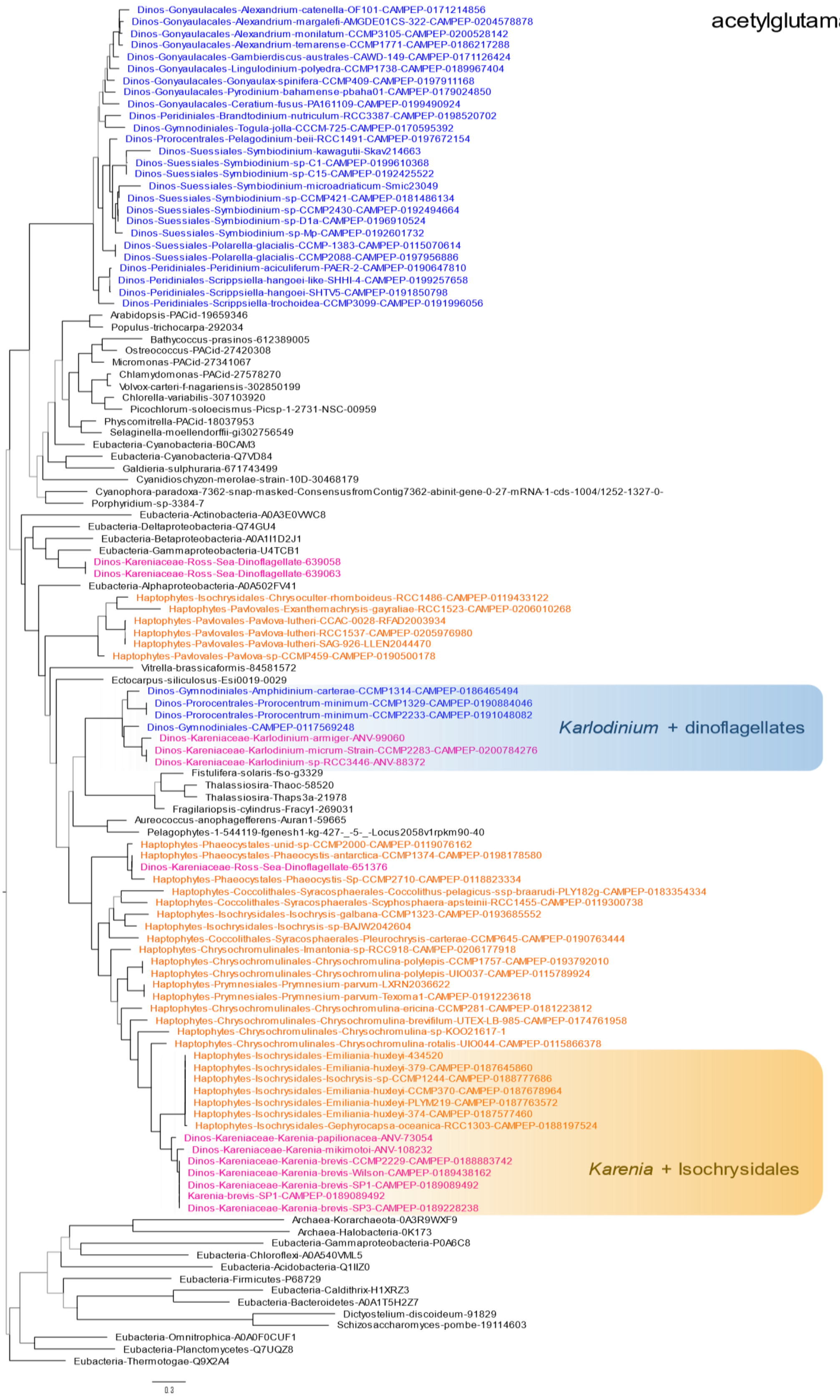

Appendix Figures S6-12: Selected phylogenetic trees of plastidial proteins with inferred green and brown algal origin; all trees are unrooted and constructed by the same pipeline as the rest of single-gene trees using all retrieved plastid-targeted kareniacean homologs as seed sequences before removing redundant hits. Bootstrap support is expressed by the branch colour (black for ≥90%, dark grey for ≥75%, light grey for <75%). Sequences from the studied kareniaceans are coloured pink with proteins with predicted plastid-targeting signal in darker shades and annotated “CP” at the end of the ID; dinoflagellates are coded blue; haptophytes are coded orange; green algae (Chloroplastida) are coded green; and brown algae (Ochrophyta) are coded brown.

Appendix Figure S6: Brown origin of plastidial threonyl-tRNA synthetase in all three genera.

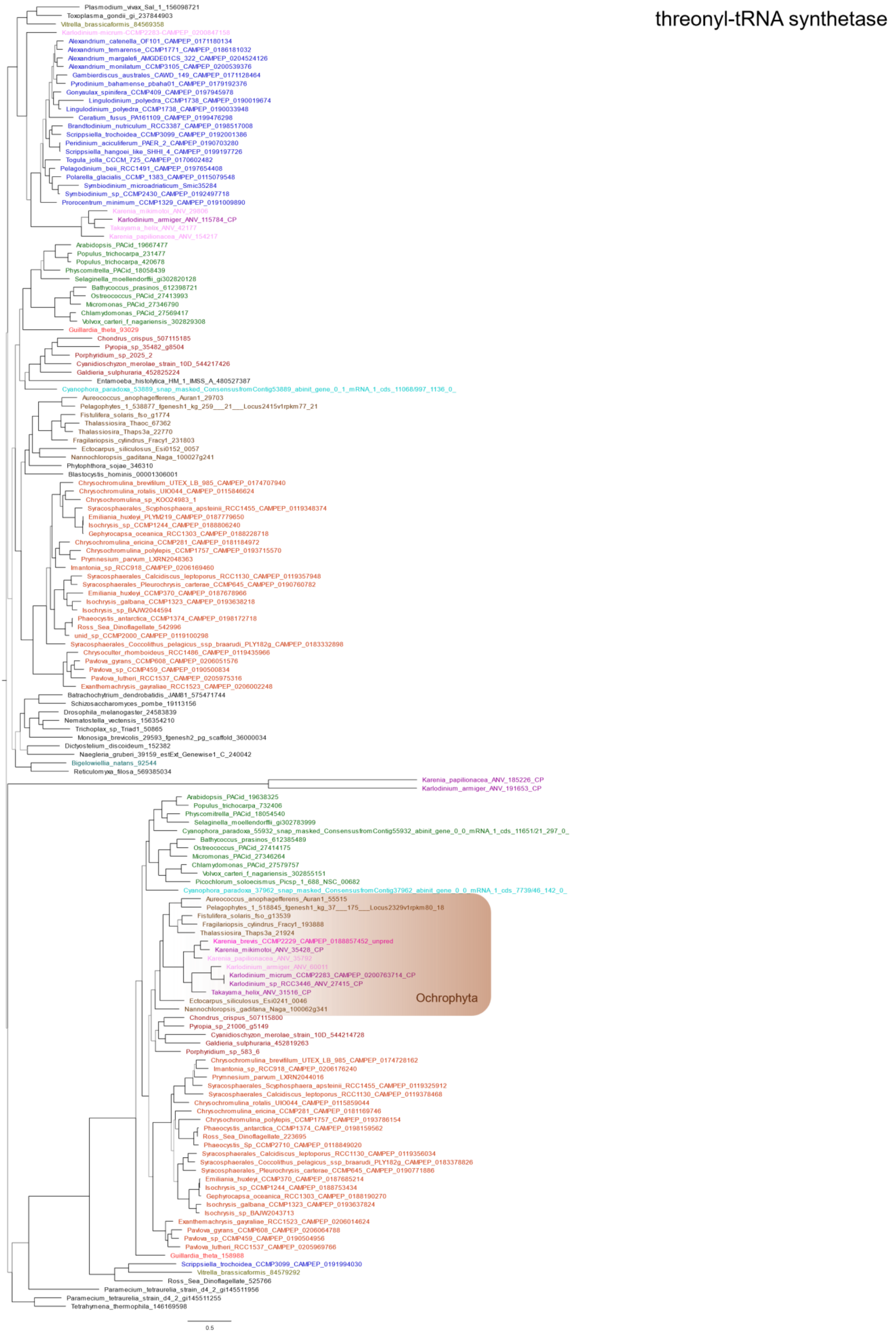

Appendix Figure S7: Green origin of plastidial prolyl-tRNA synthetase in Takayama.

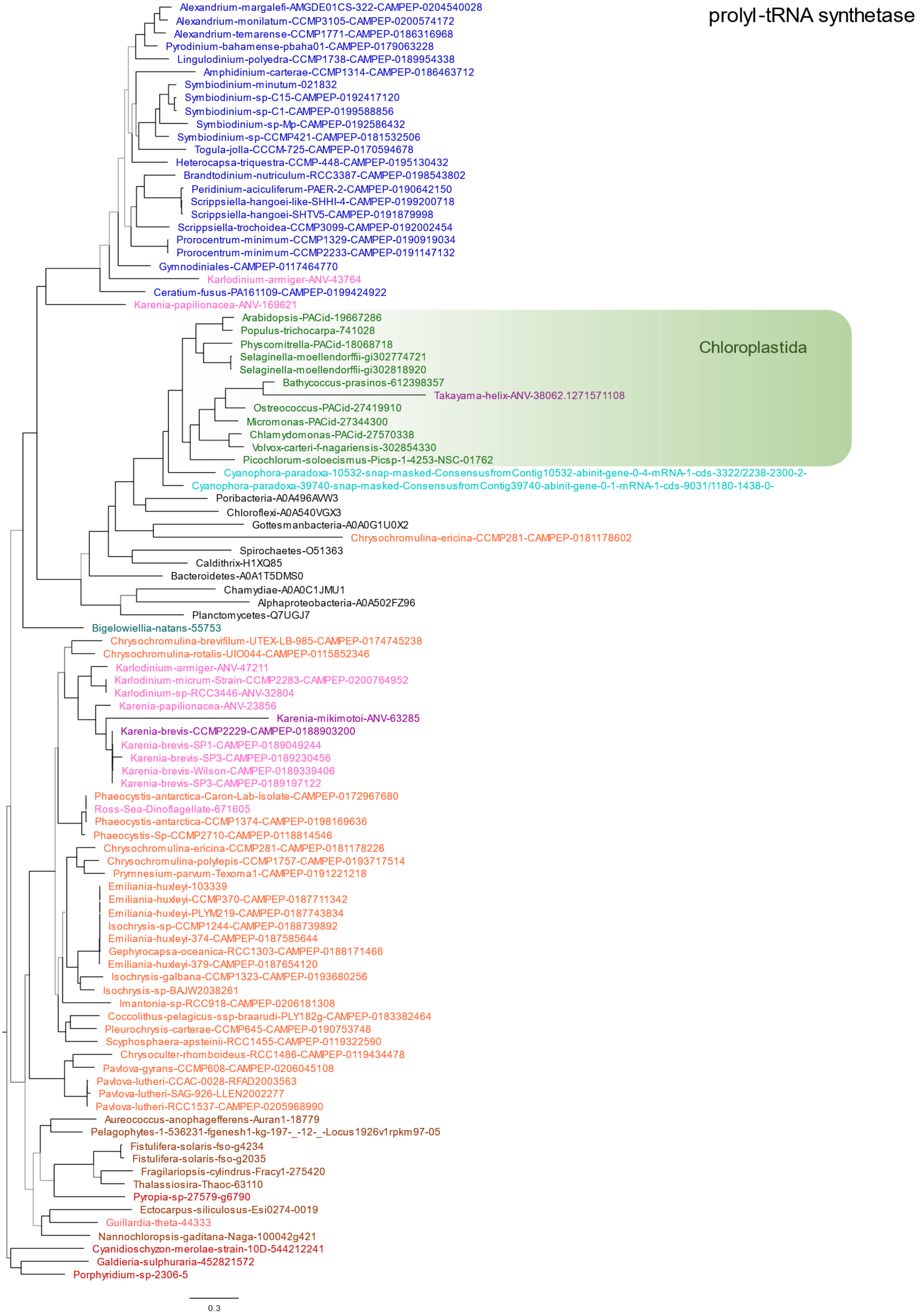

Appendix Figure S8: Brown origin of plastidial asparaginyl-tRNA synthetase in *Karenia* and *Karlodinium*.

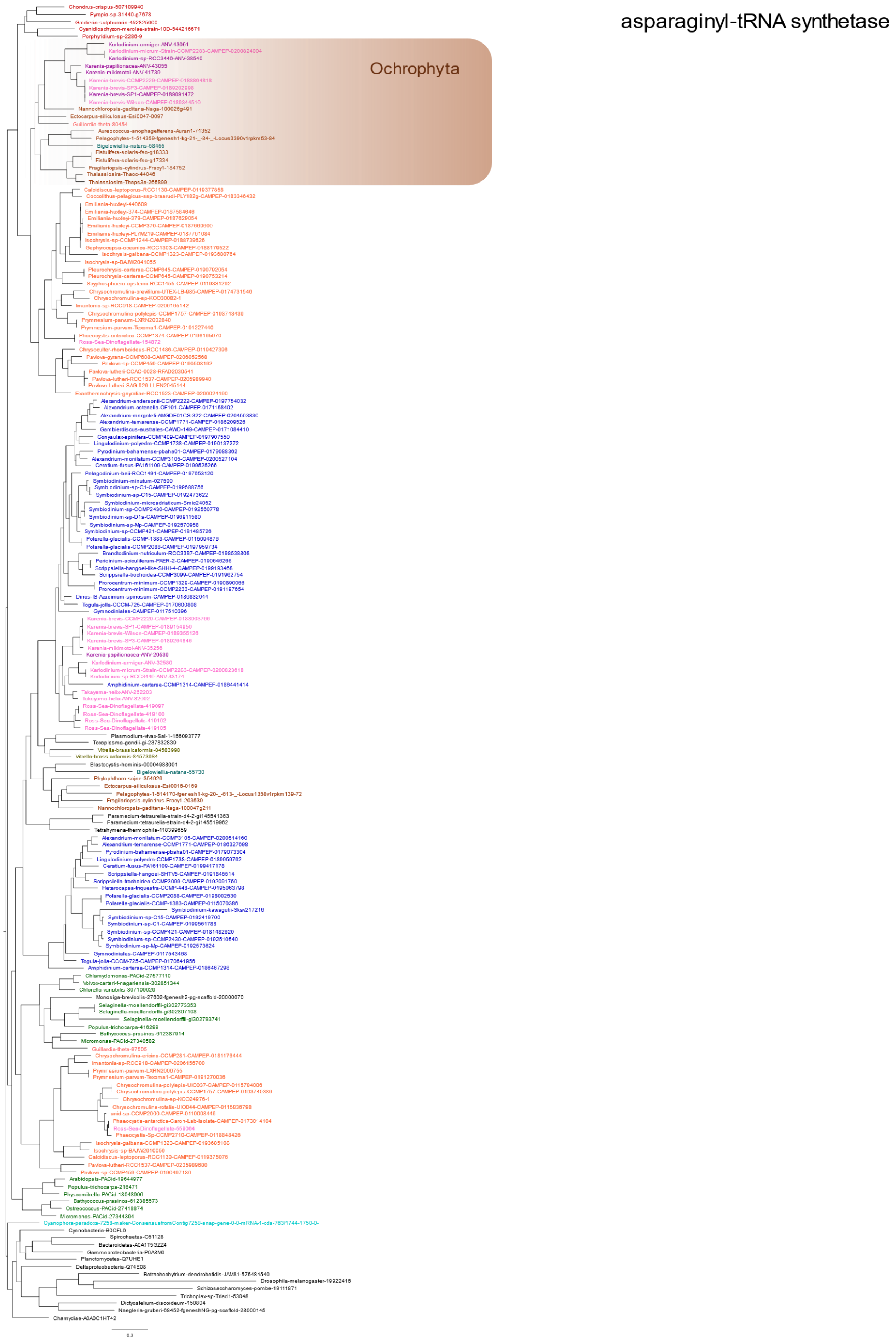

Appendix Figure S9: Green origin of sulfoquinovosyltransferase (SQD2) in all three genera.

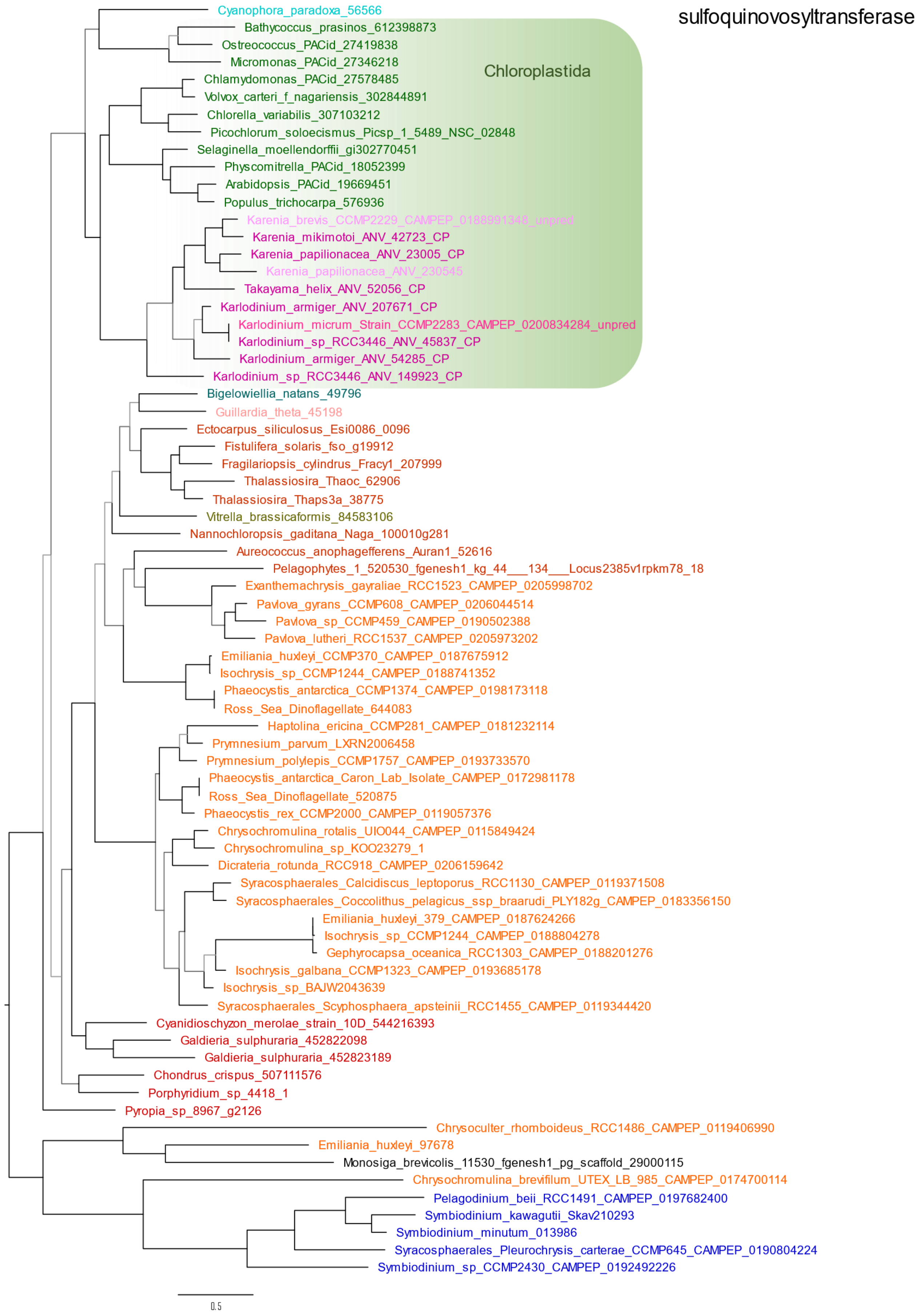

Appendix Figure S10: Brown origin of the 15-cis-phytoene synthase in all genera.

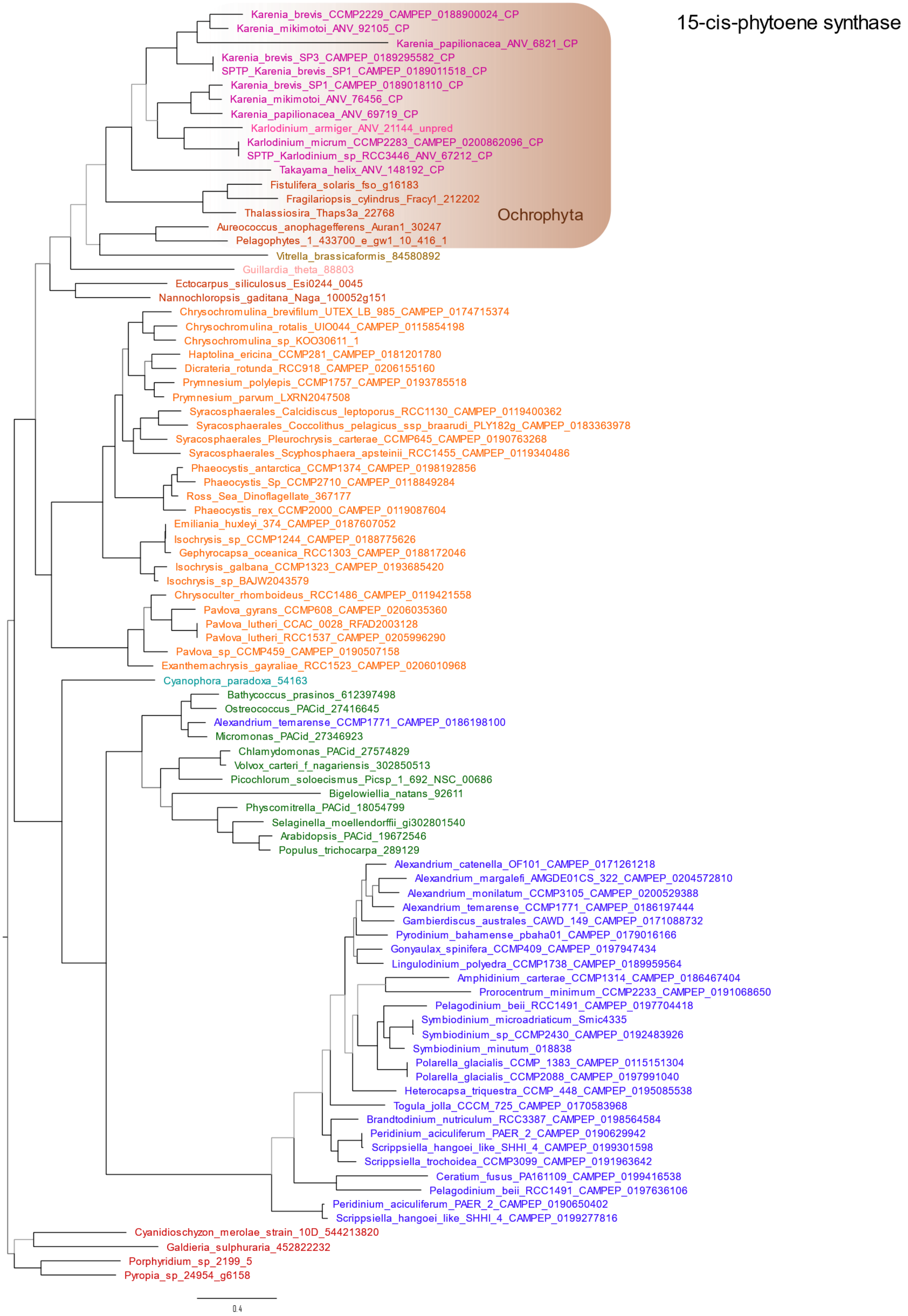

Appendix Figure S11: Green origin of the homogentisate solanesyltransferase synthase in Karenia and Karlodinium.

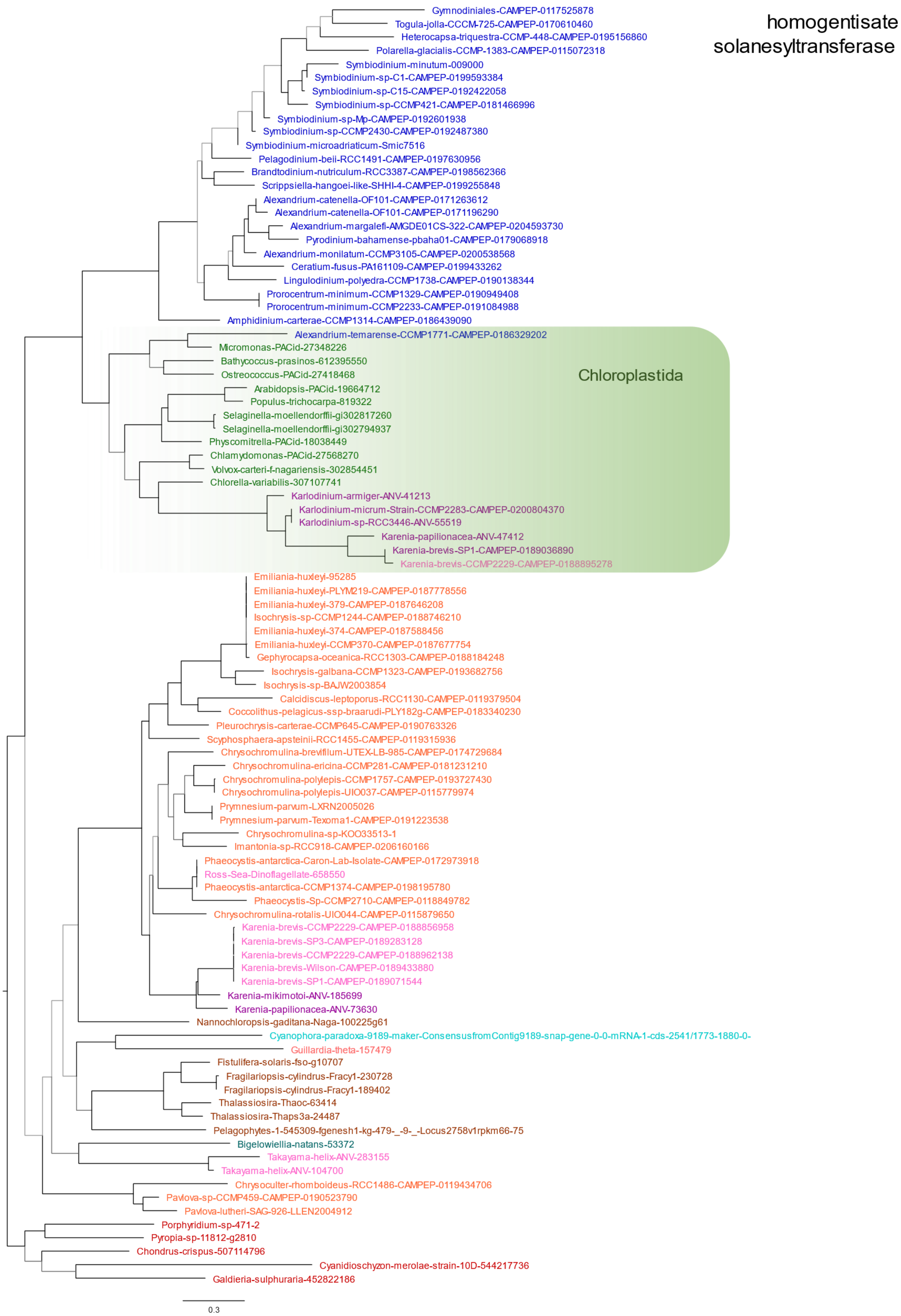

Appendix Figure S12: Brown origin of the acetyl-CoA carboxylase synthase in all three genera.

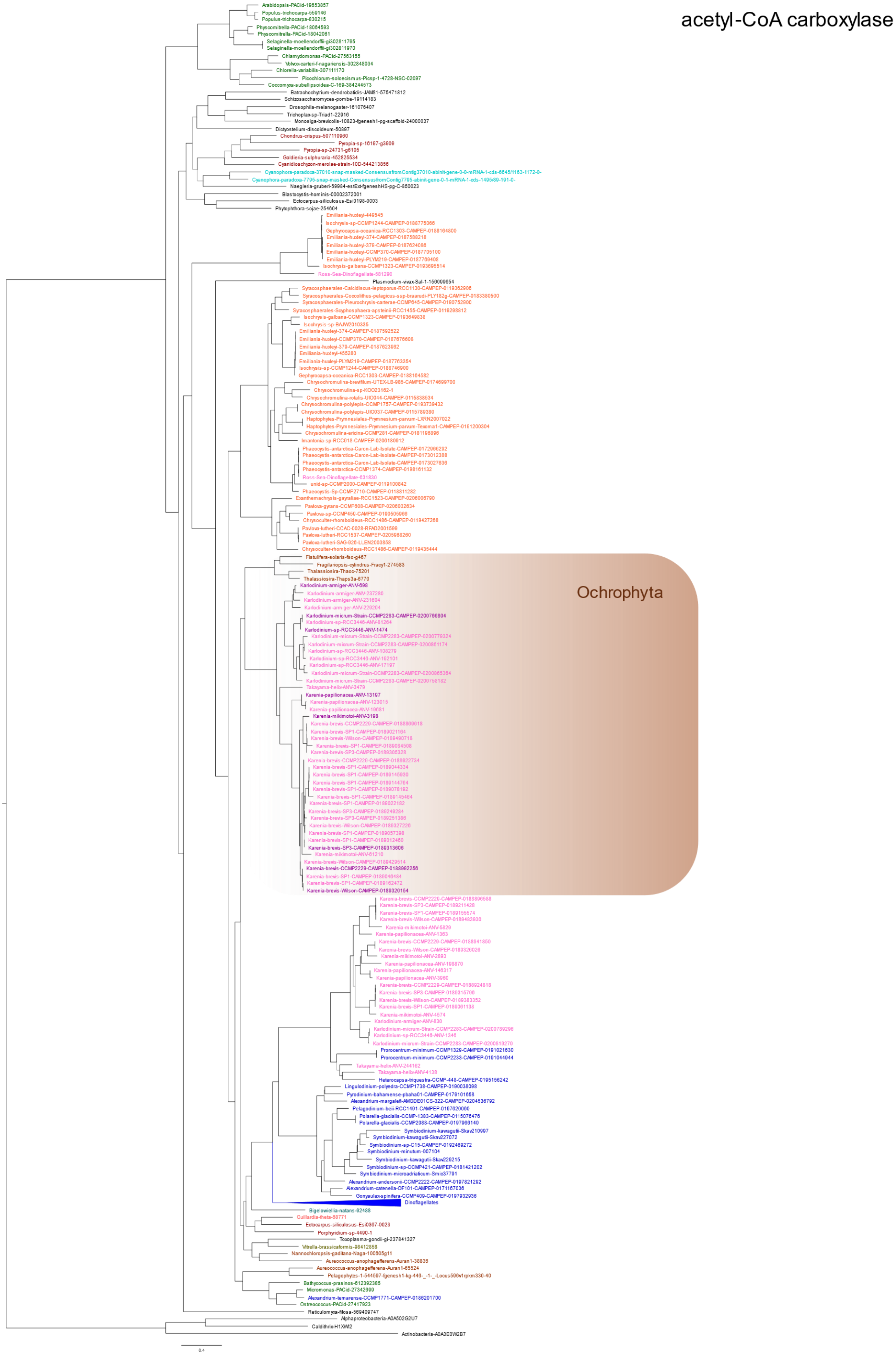

Appendix Figure S13: An alternative display of Figure 3 with additional information regarding the RSD homologs of the presented proteins shown as a drop-like shape in the bottom corner of each rosette. RSD homologs are colour-coded as per other karenicean proteins, with absence indicating that the protein was not identified or predicted as plastid-targeted.

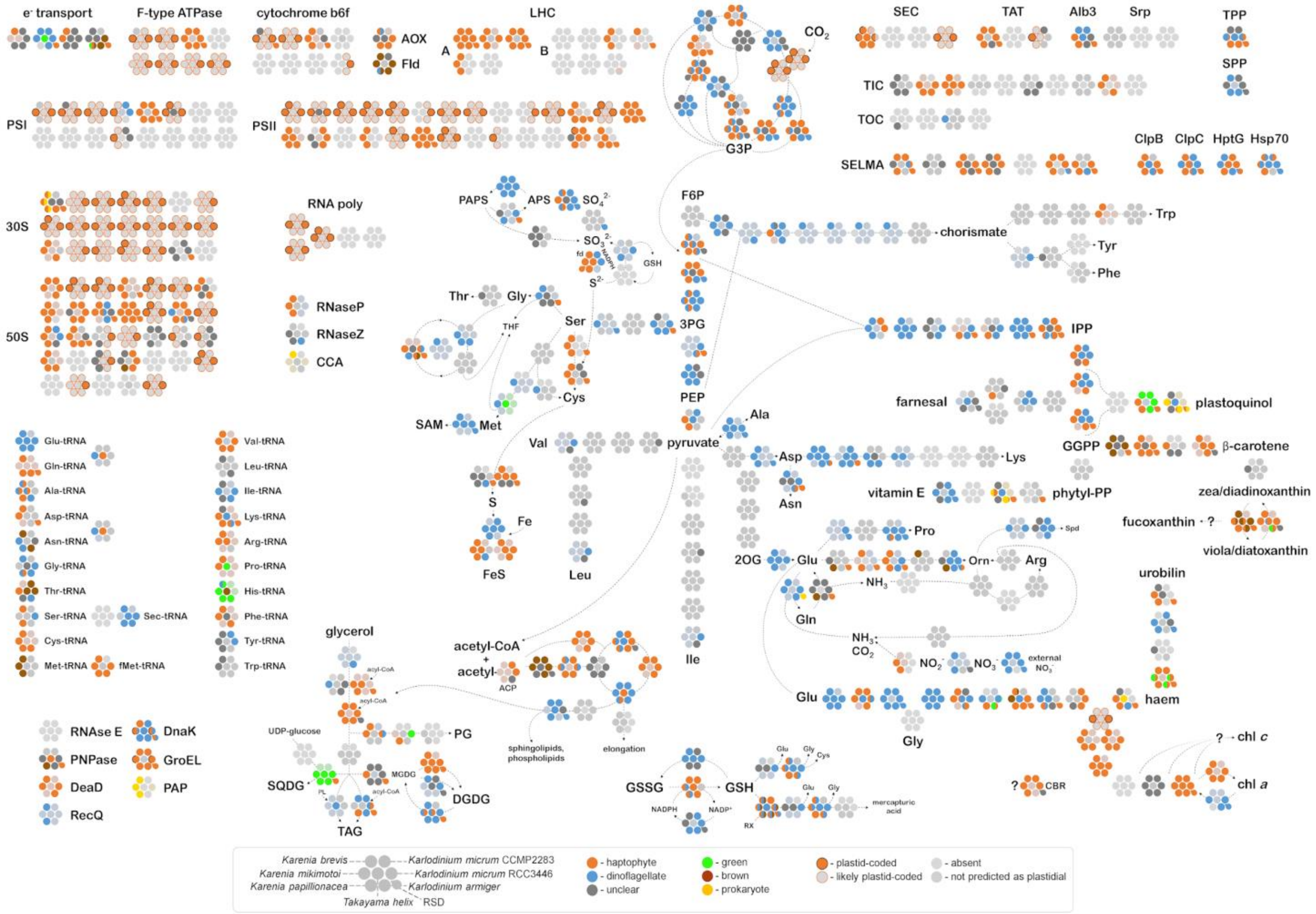

Appendix Figure S14: Amino acid composition of signal peptides of the putative plastid-targeted proteins of five peridinin dinoflagellates, five haptophytes, and plastid-early and plastid-late proteins of the seven investigated kareniaceans. Hydrophobic residues dominate with A and L more slightly more frequent in peridinin dinoflagellates and haptophytes and F and I slightly more frequent in kareniaceans. The most notable is the enrichment of C in kareniaceans, especially in plastid-late proteins, reflective of the broadly conserved LACLAC motif.

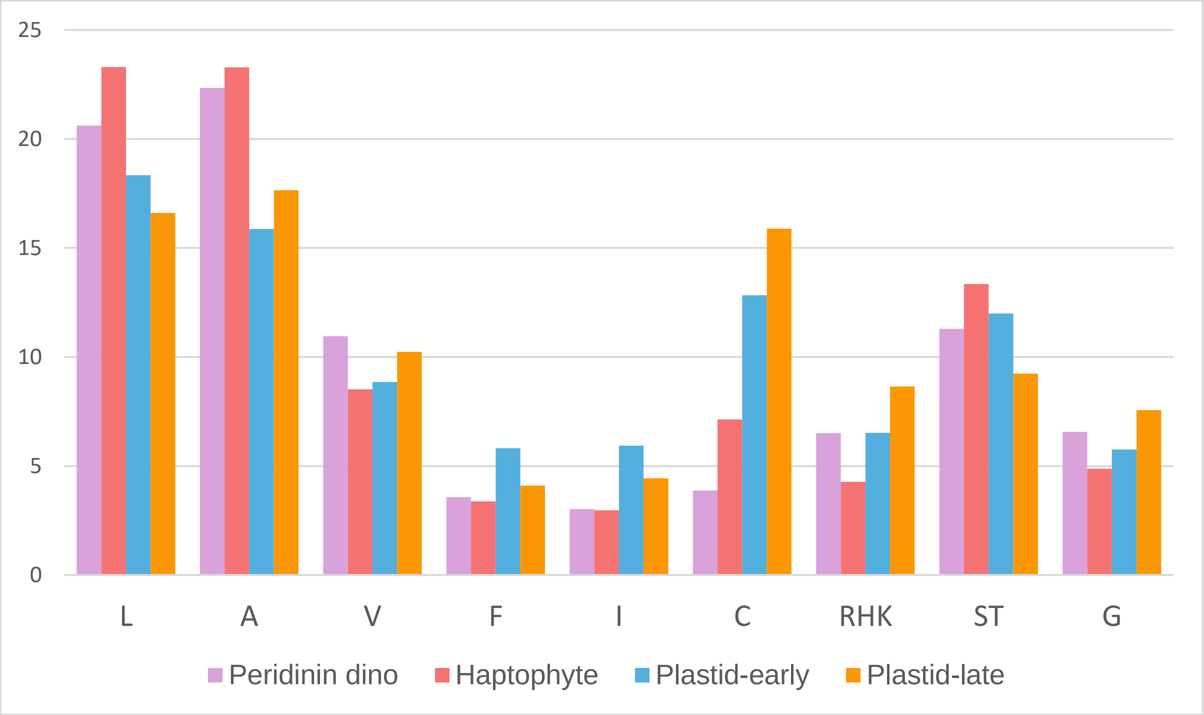

Appendix Figure S15: Sequence logos of the N-terminal domains (signal peptide and partial transit peptide) created from the full plastid protein dataset and plastid-late subset separately for thylakoid and stromal proteins. Logos based on plastid-early proteins were not considered due to the very low number of thylakoid proteins in this subset. No major differences were observed between the samples, except for a slightly higher preference for proline and glutamate residues in the non-conserved region downstream of the GRR motif in plastid-late thylakoid proteins. Presumed signal peptidase cleavage sites are shown with vertical arrows.

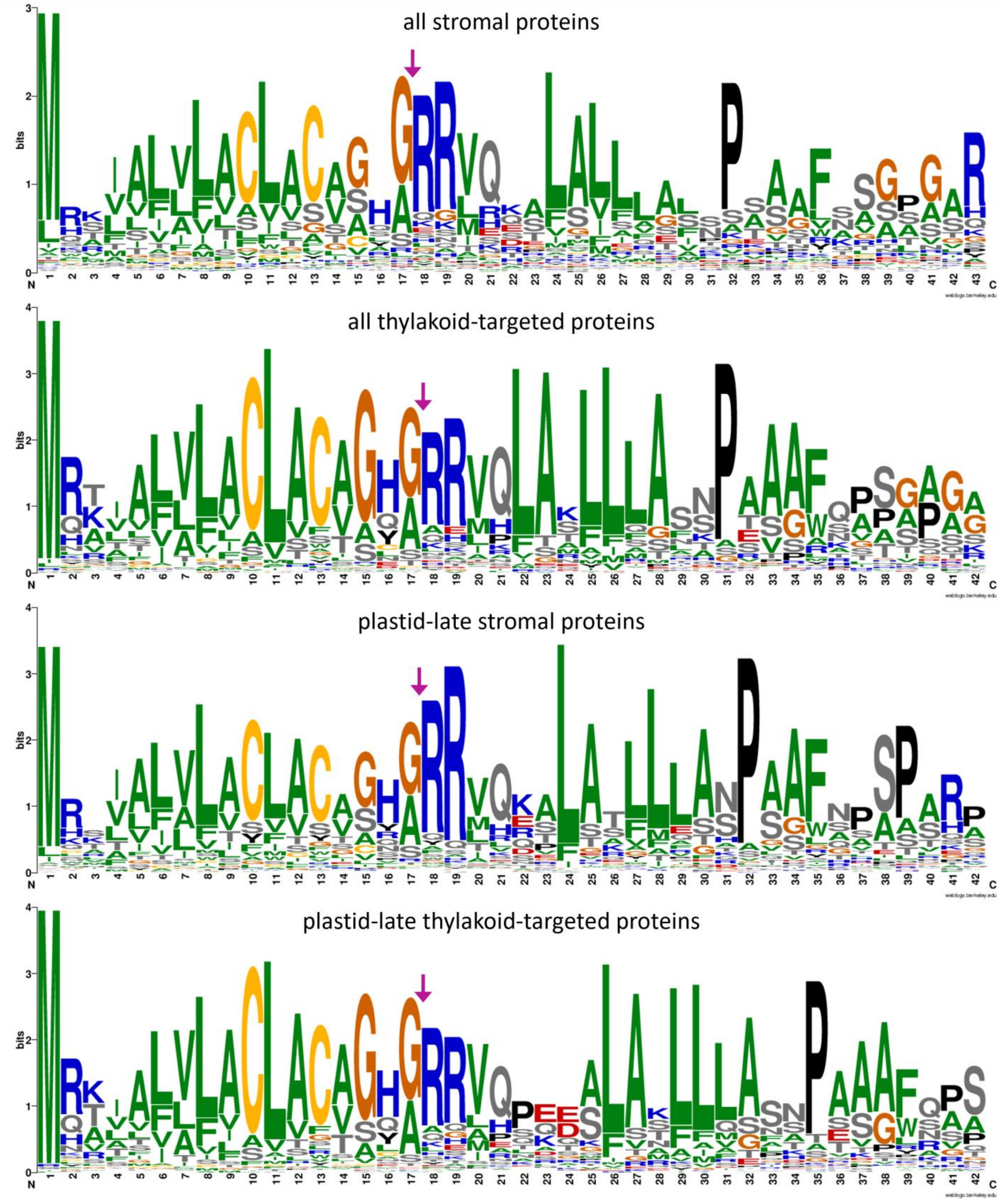

Appendix Figure S16: Partial Least Square analysis of Tara Oceans relative abundances of the three studied kareniacean genera and haptophytes correlated against various environmental parameters. While haptophytes, Karlodinium and Takayama exhibit weak negative correlations with nitrate, phosphate, and iron, Karenia shows the opposite trend. Karenia and Karlodinium both show weak positive correlations with salinity and negative correlations with silicate, while Takayama and haptophytes show the opposite trends.

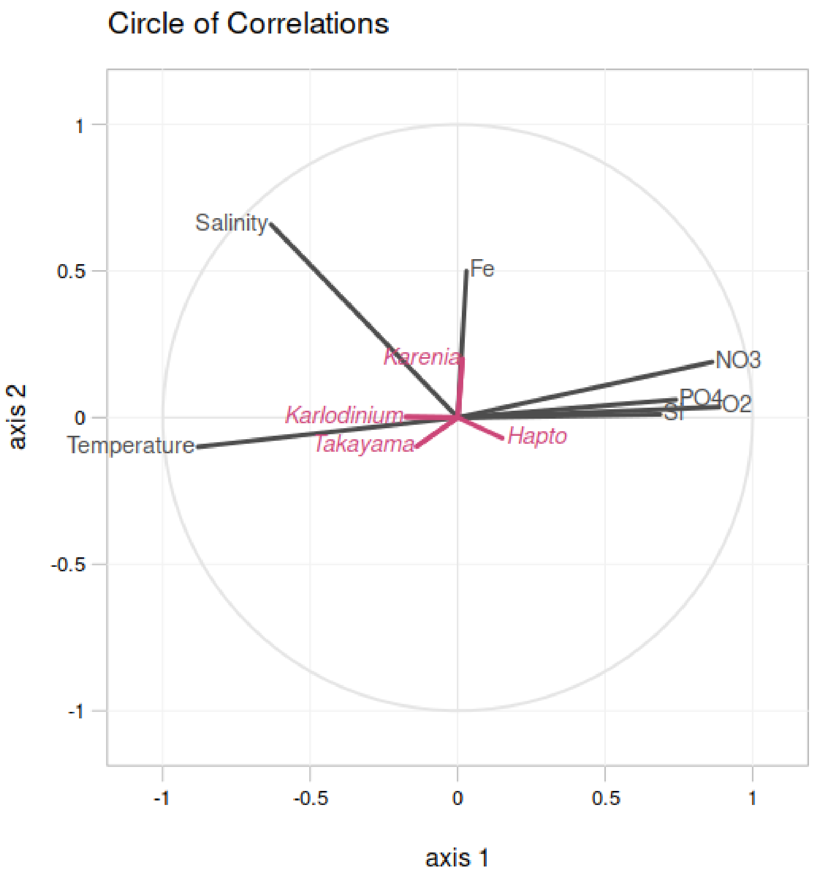

Appendix Figure S17: Correlation matrix of the three kareniacean genera abundances in Tara Oceans stations. A weak positive correlation is observed between Takayama and Karenia (p = 3.6e-05) and between Takayama and Karlodinium (p = 8.1e-03), but no significant correlation is found between Karenia and Karlodinium.

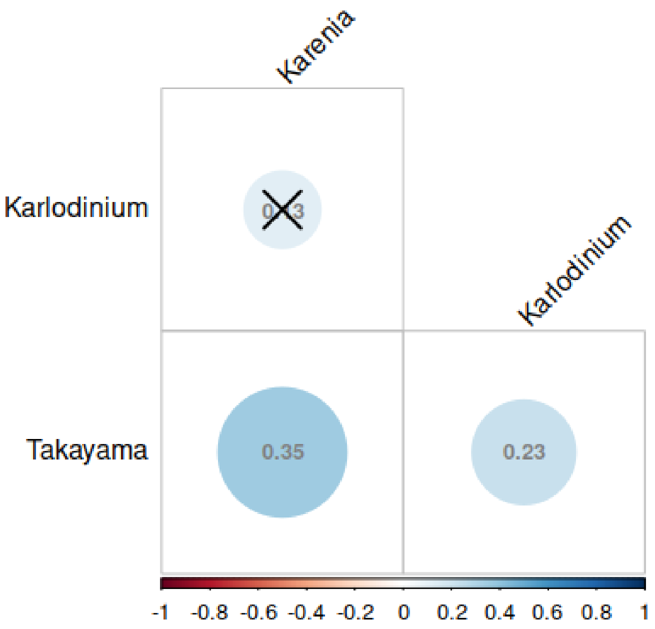

Appendix Figure S18: Sequence logos of the N-terminal region of the model plastid-targeted proteins for Karenia brevis and Karlodinium micrum based on which the scoring matrix was prepared. The signal peptide cleavage position is between positions 10 and 11.

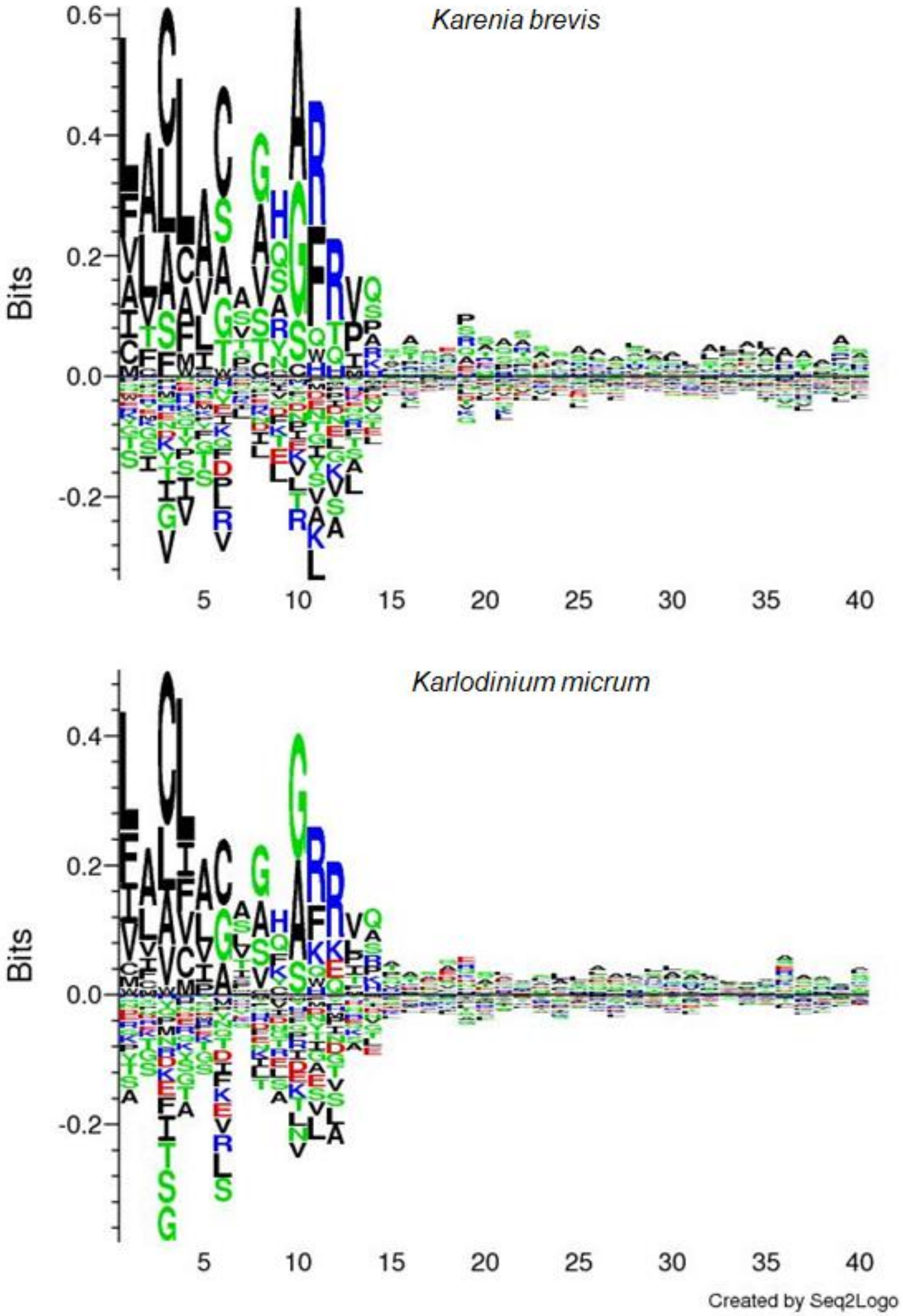

Appendix Figure S19: The effect of SignalP 3.0 score threshold modification on the specificity/ sensitivity of prediction on model kareniacean datasets. The plot shows the ratio of proteins in the plastid (positive, blue) and non-plastid (negative, red) training datasets (Dataset EV6) that pass prediction by modified ASAFind with different SignalP cleavage site score thresholds. Based on the difference between the two ratios (subtraction, grey) the best sensitivity/ specificity ratio is achieved with a -10 modification of the default threshold.

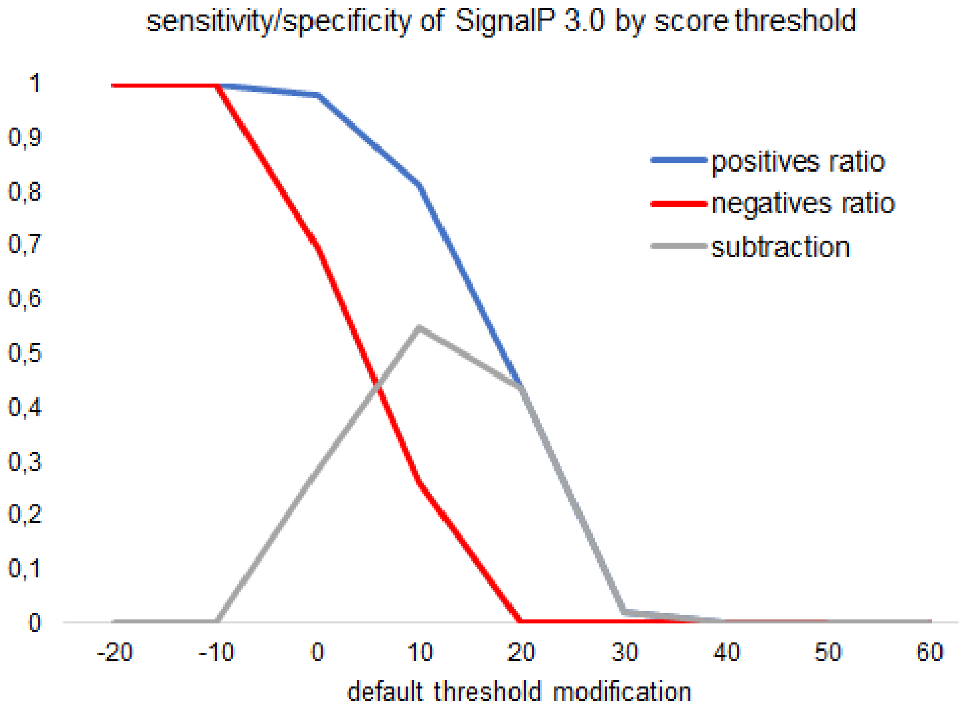

Appendix Figure S20: Sensitivity and specificity of the prediction software and their combinations tested during prediction optimization. While all methods exhibit comparable specificity, the sensitivity achieved by the modified ASAFind script is much higher, particularly in conjunction with SignalP 5.0.

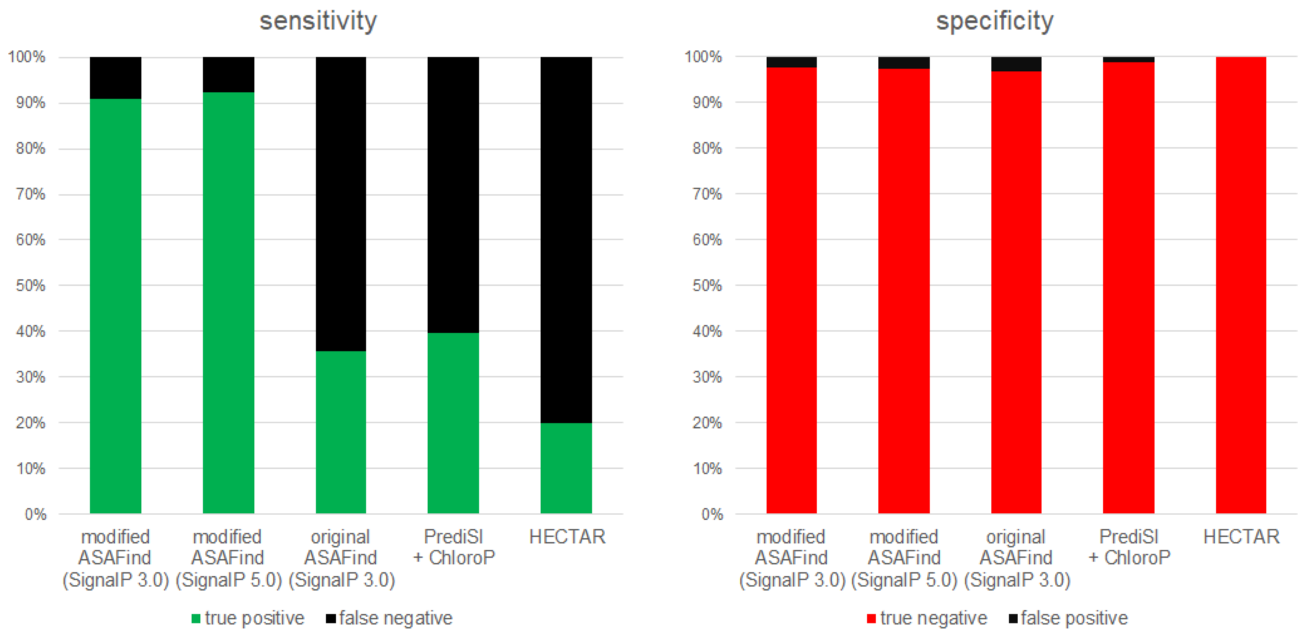

Appendix Figure S21: Length distributions across the 29,304 alignments used for single-gene tree building. 90% of the alignments were 137-736 positions long; with a median value of 305.

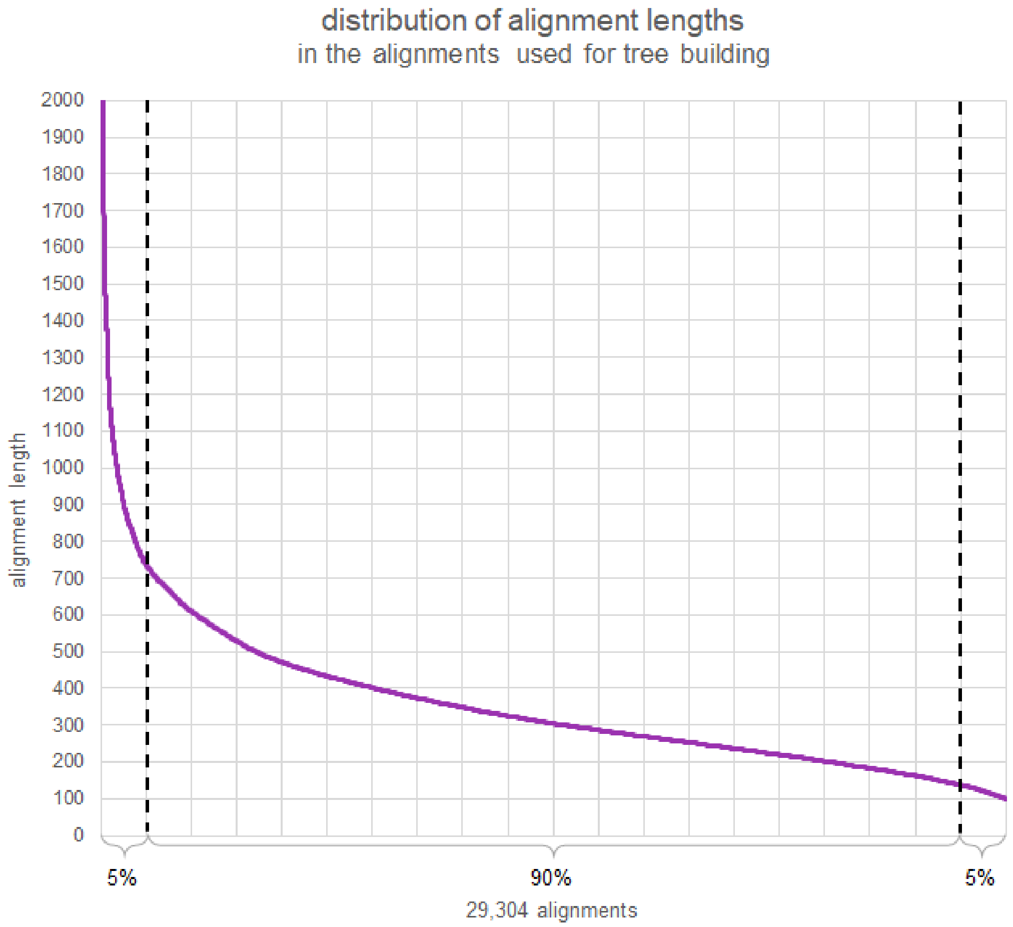

Appendix Figure S22: Dataset size distribution across the 29,304 alignments used for single-gene tree building. 90% of the alignments contained 16-171 sequences; with a median value of 74.

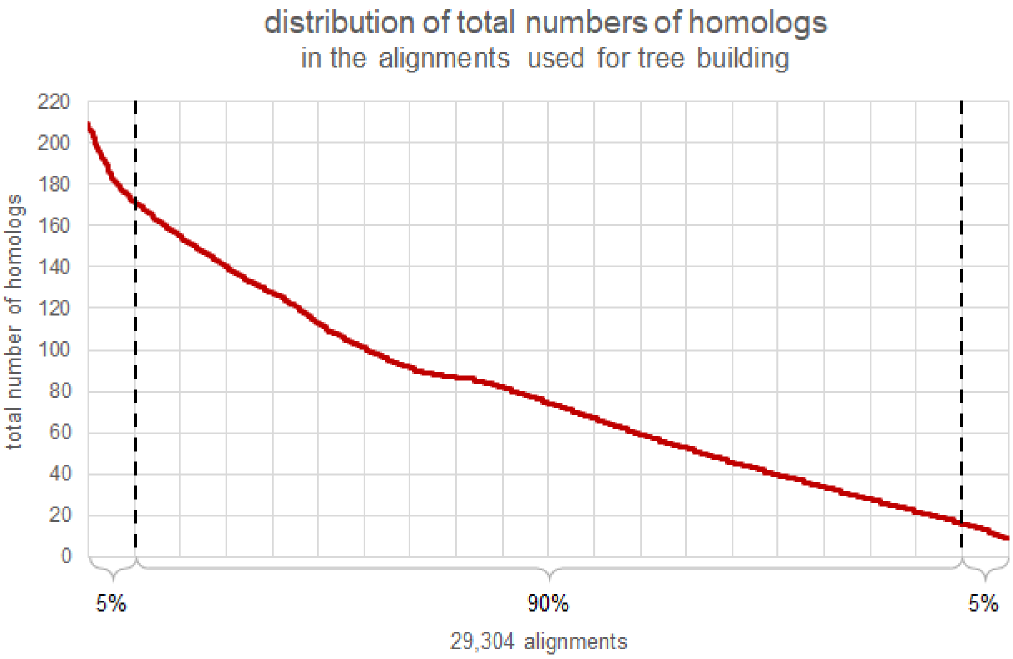

Appendix Figure S23: Average gap percentage distribution across the 29,304 alignments used for single-gene tree building. 90% of the alignments contained 1.1-24.5% of gaps; with a median value of 6.6%.

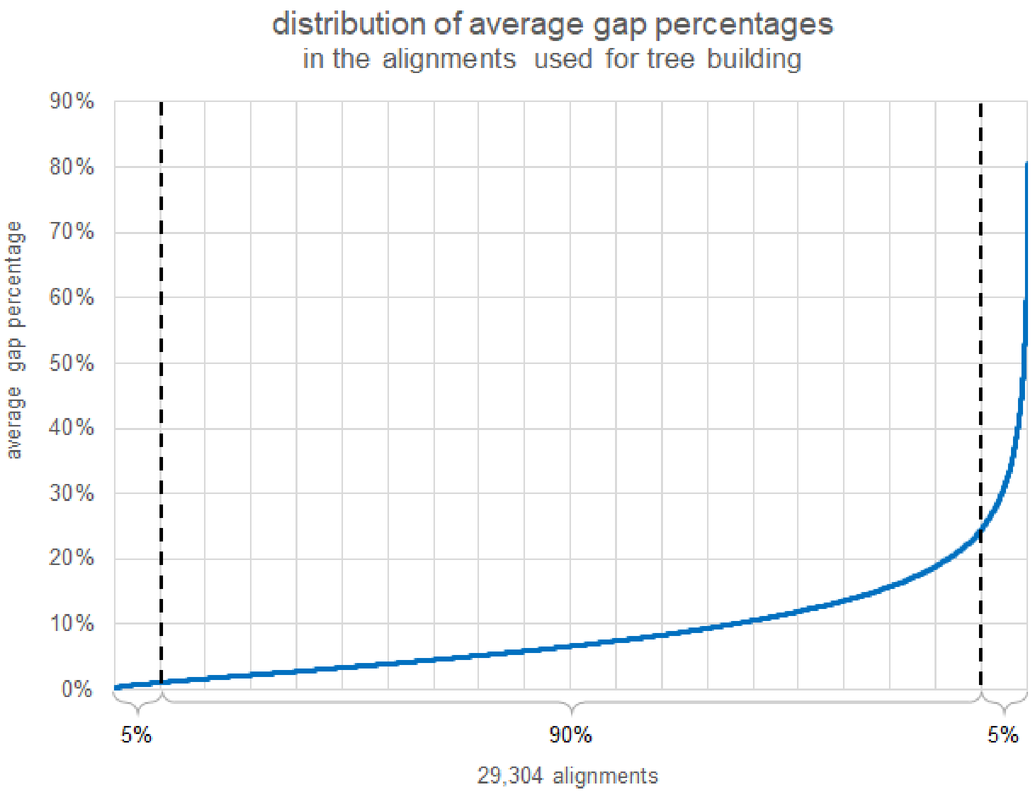

Supplement: Supplementary file 1 — Appendix [file 44319_2024_103_MOESM1_ESM.pdf]
