# Supplementary figures and images for: New plastids, old proteins: repeated endosymbiotic acquisitions in kareniacean dinoflagellates
Source: EMBO Rep. 2024 Mar 18;25(4):16. doi: 10.1038/s44319-024-00103-y (PMC11014865; doi:10.1038/s44319-024-00103-y)

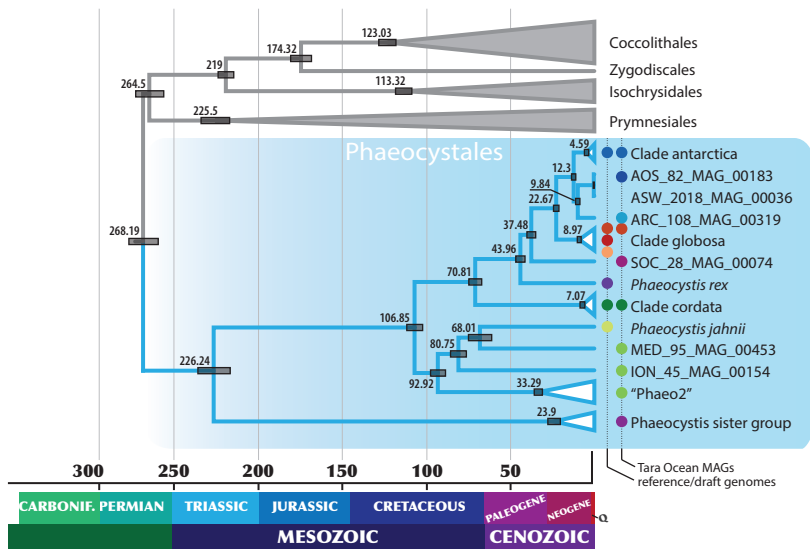

Supplement: Supplementary file 11 — Dataset EV10 [file 44319_2024_103_MOESM11_ESM.zip › Dataset EV10/plastid_genome_phylogeny_files/16S/alternative alignments and trees 2/Phaecystales.pdf]

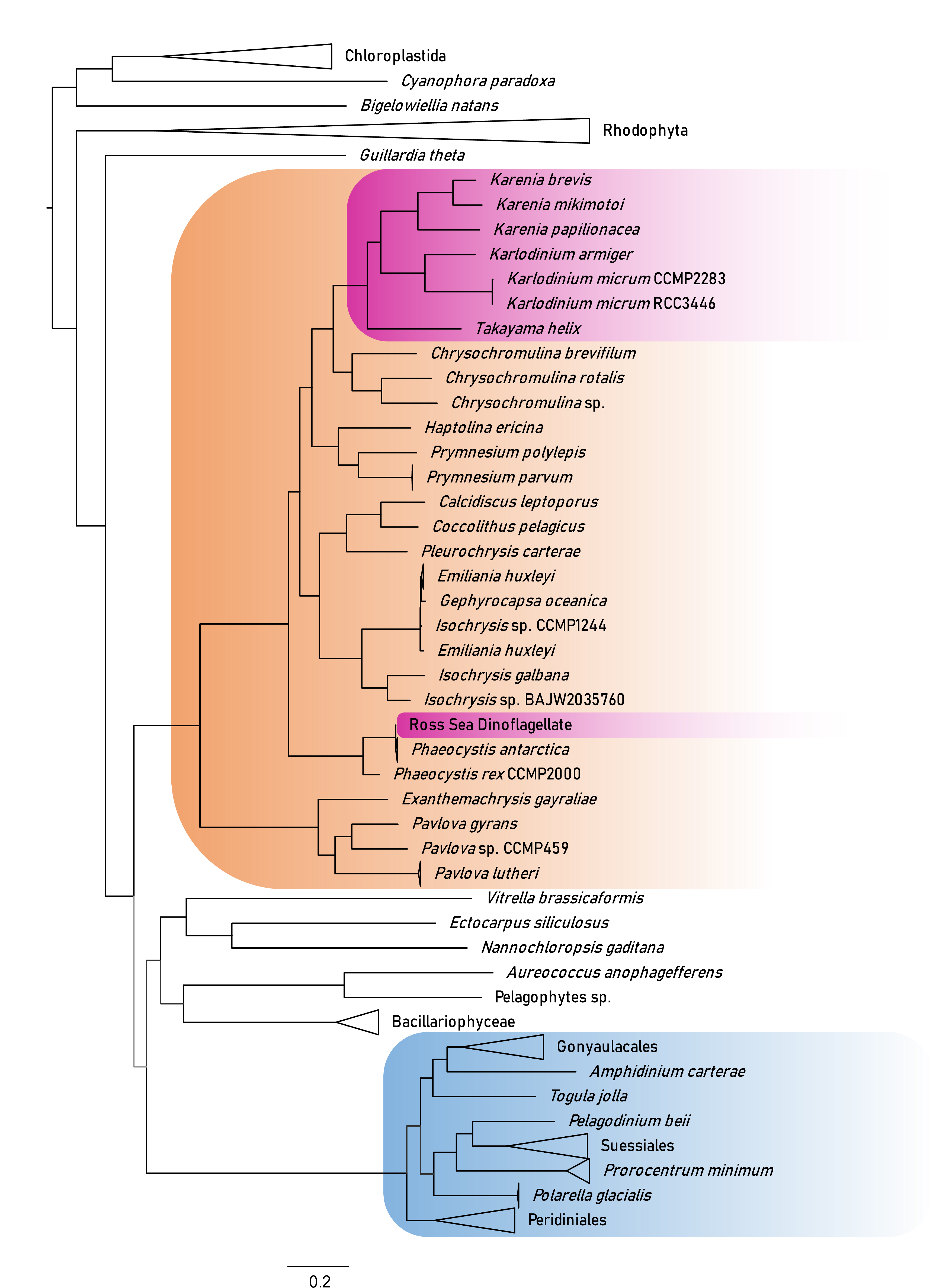

Supplement: Supplementary file 11 — Dataset EV10 [file 44319_2024_103_MOESM11_ESM.zip › Dataset EV10/plastid_protein_phylogeny_files/concatenations/full concatenation/tree.png]
