# Supplementary material for: New plastids, old proteins: repeated endosymbiotic acquisitions in kareniacean dinoflagellates
Source: EMBO Rep. 2024 Mar 18;25(4):16. doi: 10.1038/s44319-024-00103-y (PMC11014865; doi:10.1038/s44319-024-00103-y)
Supplement: Supplementary file 14 — Expanded View Figures [file 44319_2024_103_MOESM14_ESM.pdf]

Expanded View Figures

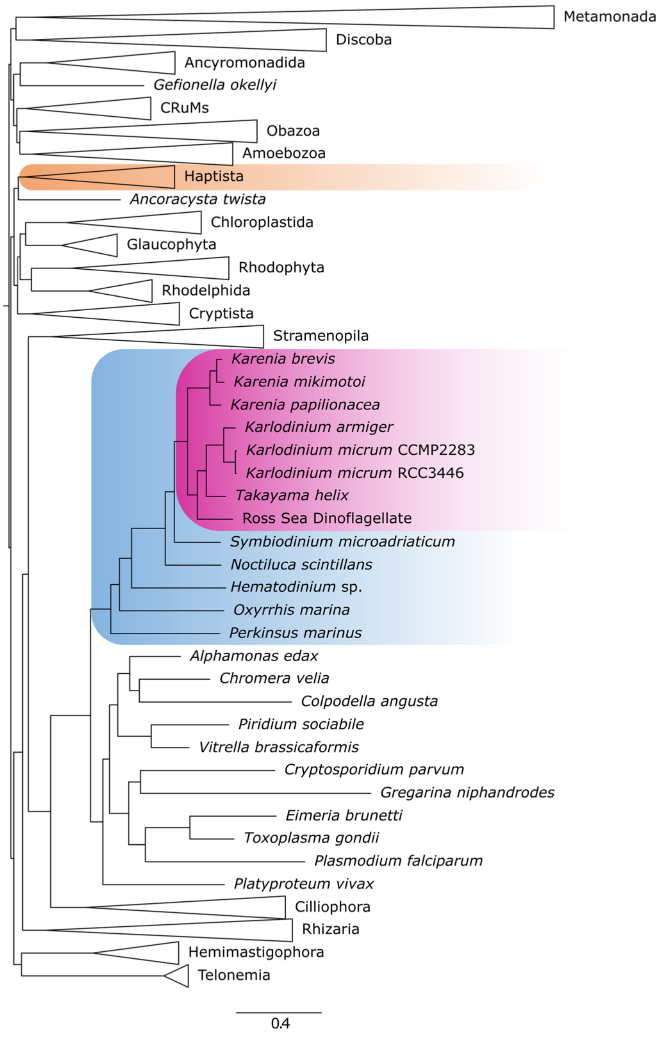

Figure EV1. The eight kareniaceans in the pan-eukaryotic phylogenetic context as reconstructed by IQ-TREE based on a matrix of 241 genes prepared using PhyloFisher toolkit.

The inner relationships between the studied kareniaceans are resolved with maximum support and confirm the results of previous phylogenetic analyses based on ribosomal subunits (Takahashi et al, 2019; Ok et al, 2021).

 © The Author(s)

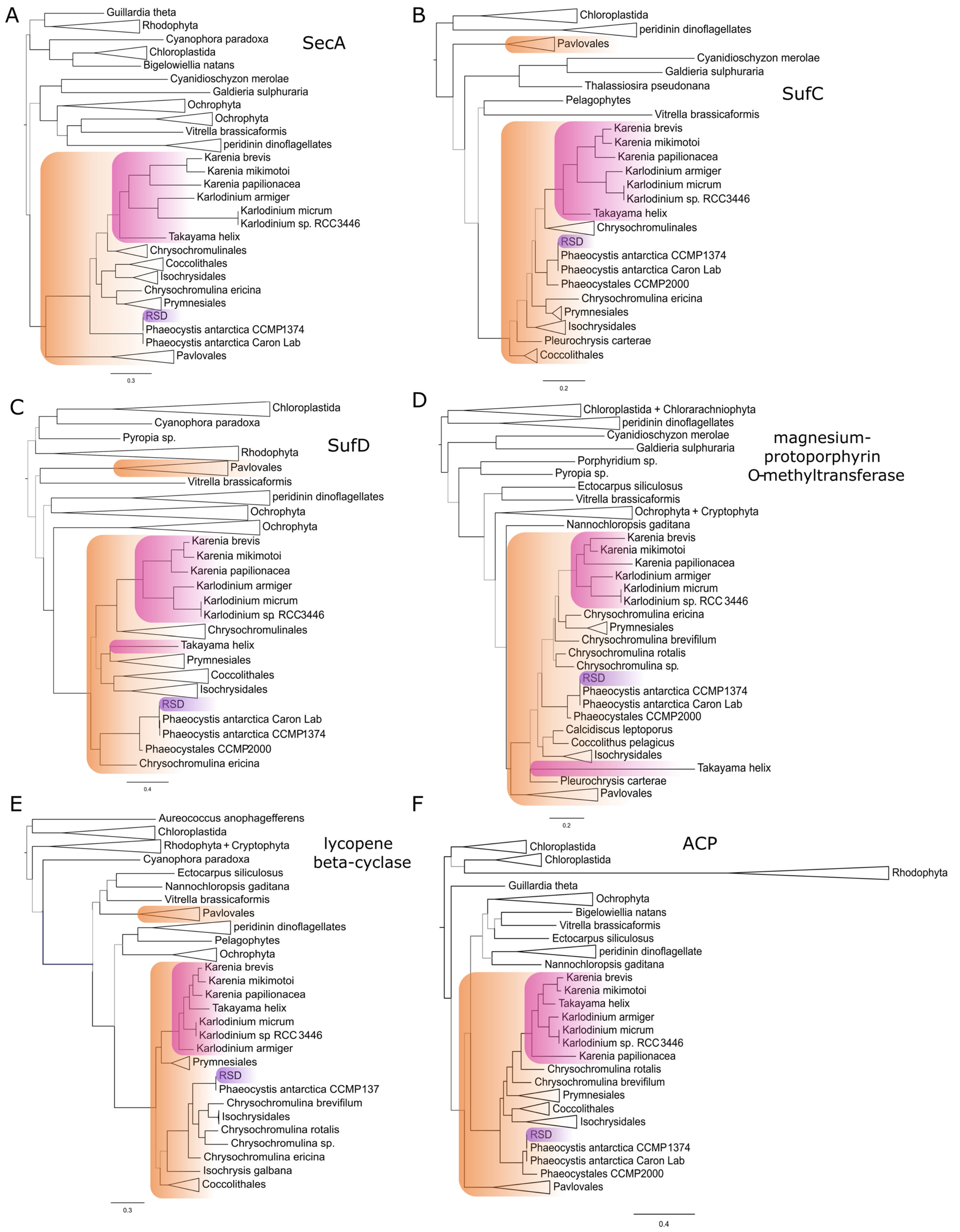
A
SecA
Guillardia theta
Rhodophyta
Cyanophora paradoxa
Chloroplastida
Bigelowiella natans
Cyanidioschyzon merolae
Galdieria sulphuraria
Ochrophyta
Ochrophyta
Vitrella brassicaformis
peridinin dinoflagellates
Karenia brevis
Karenia mikimotoi
Karenia papilionacea
Karlodinium armiger
Karlodinium micrum
Karlodinium sp. RCC3446
Takayama helix
Chrysochromulinales
Coccolithales
Isochrysidales
Chrysochromulina ericina
Prymnesiales
RSD
Phaeocystis antarctica CCMP1374
Phaeocystis antarctica Caron Lab
Pavlovales
0.3
B
SufC
Chloroplastida
peridinin dinoflagellates
Pavlovales
Cyanidioschyzon merolae
Galdieria sulphuraria
Thalassiosira pseudonana
Pelagophytes
Vitrella brassicaformis
Karenia brevis
Karenia mikimotoi
Karenia papilionacea
Karlodinium armiger
Karlodinium micrum
Karlodinium sp. RCC3446
Takayama helix
Chrysochromulinales
RSD
Phaeocystis antarctica CCMP1374
Phaeocystis antarctica Caron Lab
Phaeocystales CCMP2000
Chrysochromulina ericina
Prymnesiales
Isochrysidales
Pleurochrysis carterae
Coccolithales
0.2
C
SufD
Chloroplastida
Cyanophora paradoxa
Pyropia sp.
Rhodophyta
Pavlovales
Vitrella brassicaformis
peridinin dinoflagellates
Ochrophyta
Ochrophyta
Karenia brevis
Karenia mikimotoi
Karenia papilionacea
Karlodinium armiger
Karlodinium micrum
Karlodinium sp RCC3446
Chrysochromulinales
Takayama helix
Prymnesiales
Coccolithales
Isochrysidales
RSD
Phaeocystis antarctica Caron Lab
Phaeocystis antarctica CCMP1374
Phaeocystales CCMP2000
Chrysochromulina ericina
0.4
D
magnesium-protoporphyrin O-methyltransferase
Chloroplastida + Chlorarachniophyta
peridinin dinoflagellates
Cyanidioschyzon merolae
Galdieria sulphuraria
Porphyridium sp.
Pyropia sp.
Ectocarpus siliculosus
Vitrella brassicaformis
Ochrophyta + Cryptophyta
Nannochloropsis gaditana
Karenia brevis
Karenia mikimotoi
Karenia papilionacea
Karlodinium armiger
Karlodinium micrum
Karlodinium sp. RCC3446
Chrysochromulina ericina
Prymnesiales
Chrysochromulina brevifilum
Chrysochromulina rotalis
Chrysochromulina sp.
RSD
Phaeocystis antarctica CCMP1374
Phaeocystis antarctica Caron Lab
Phaeocystales CCMP2000
Calcidiscus leptoporus
Coccolithus pelagicus
Isochrysidales
Takayama helix
Pleurochrysis carterae
Pavlovales
0.2
E
lycopene beta-cyclase
Aureococcus anophagefferens
Chloroplastida
Rhodophyta + Cryptophyta
Cyanophora paradoxa
Ectocarpus siliculosus
Nannochloropsis gaditana
Vitrella brassicaformis
Pavlovales
peridinin dinoflagellates
Pelagophytes
Ochrophyta
Karenia brevis
Karenia mikimotoi
Karenia papilionacea
Takayama helix
Karlodinium micrum
Karlodinium sp RCC3446
Karlodinium armiger
Prymnesiales
RSD
Phaeocystis antarctica CCMP137
Chrysochromulina brevifilum
Isochrysidales
Chrysochromulina rotalis
Chrysochromulina sp.
Chrysochromulina ericina
Isochrysis galbana
Coccolithales
0.3
F
ACP
Chloroplastida
Chloroplastida
Rhodophyta
Guillardia theta
Ochrophyta
Bigelowiella natans
Vitrella brassicaformis
Ectocarpus siliculosus
peridinin dinoflagellate
Nannochloropsis gaditana
Karenia brevis
Karenia mikimotoi
Takayama helix
Karlodinium armiger
Karlodinium micrum
Karlodinium sp. RCC3446
Karenia papilionacea
Chrysochromulina rotalis
Chrysochromulina brevifilum
Prymnesiales
Coccolithales
Isochrysidales
RSD
Phaeocystis antarctica CCMP1374
Phaeocystis antarctica Caron Lab
Phaeocystales CCMP2000
Pavlovales
0.4

◀ **Figure EV2. Example single-gene trees for proteins of plastid-late origin, with specifically plastidial function and present in all studied organisms.**

The non-RSD kareniacean sequences typically resolve adjacent to Chrysochromulinales or a mixed Chrysochromulinales/Prymnesiales clade. Their inner topology varies and typically differs from the organismal one (Figure EV1), with *Takayama* often branching as sister to the rest (**A**, **B**) and sometimes even separately (**C**, **D**), albeit with low support, or inside the *Karenia/Karlodinium* clade but sister to *Karenia* rather than *Karlodinium* (**E**, **F**). All species of *Karenia* and *Karlodinium* are, however, consistently retrieved as monophyletic, suggesting a common chrysochromulinalean-like origin of their nucleus-encoded plastid protein inventory, which is for the most part, also shared with *Takayama*.

 © The Author(s)

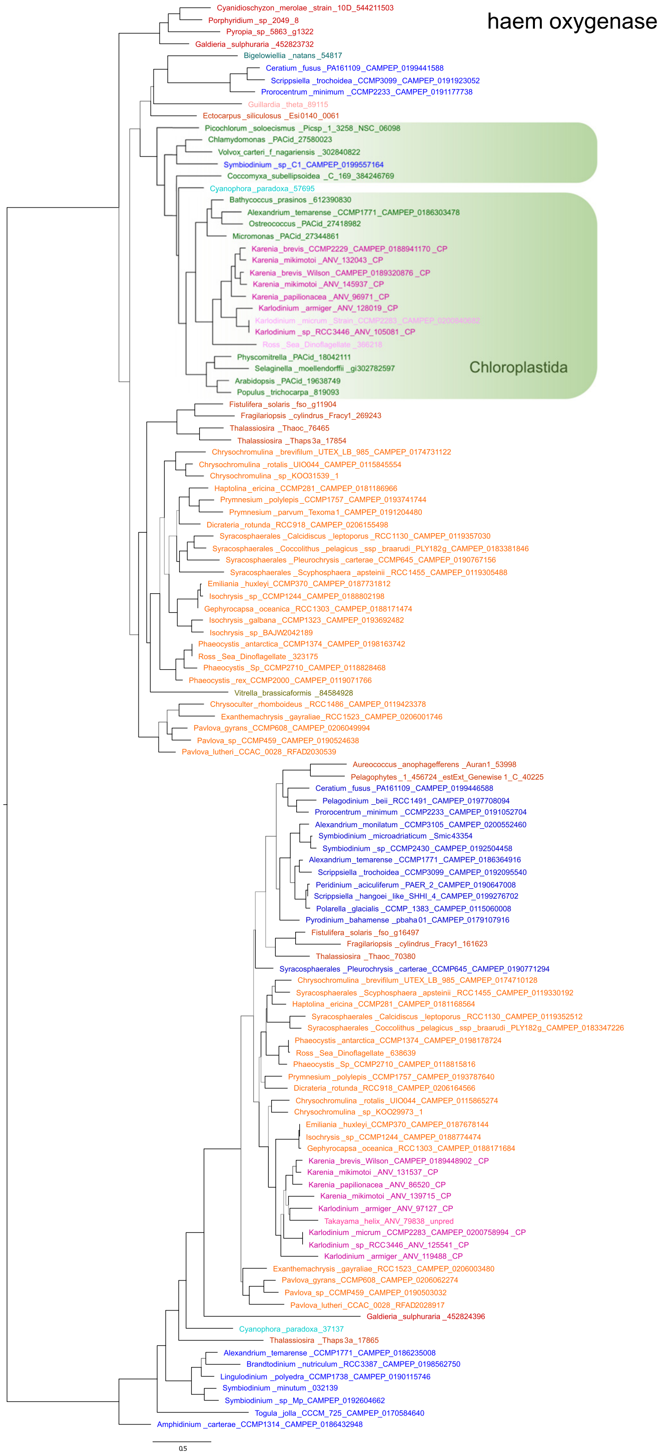

Figure EV3. Automatically generated phylogenetic tree showing the green origin of one form of heme oxygenase in *Karenia*, *Karlodinium*, and the RSD (no *Takayama* homolog was retrieved); the second form is of plastid-late origin in all genera.

Bootstrap support is expressed by the branch color (black for ≥90%, dark gray for ≥75%, and light gray for <75%). Kareniacean sequences are colored pink with proteins with predicted plastid-targeting signal in darker shades and annotated "CP" suffix; dinoflagellates are coded blue; haptophytes are coded orange; green algae (Chloroplastida) are coded green.

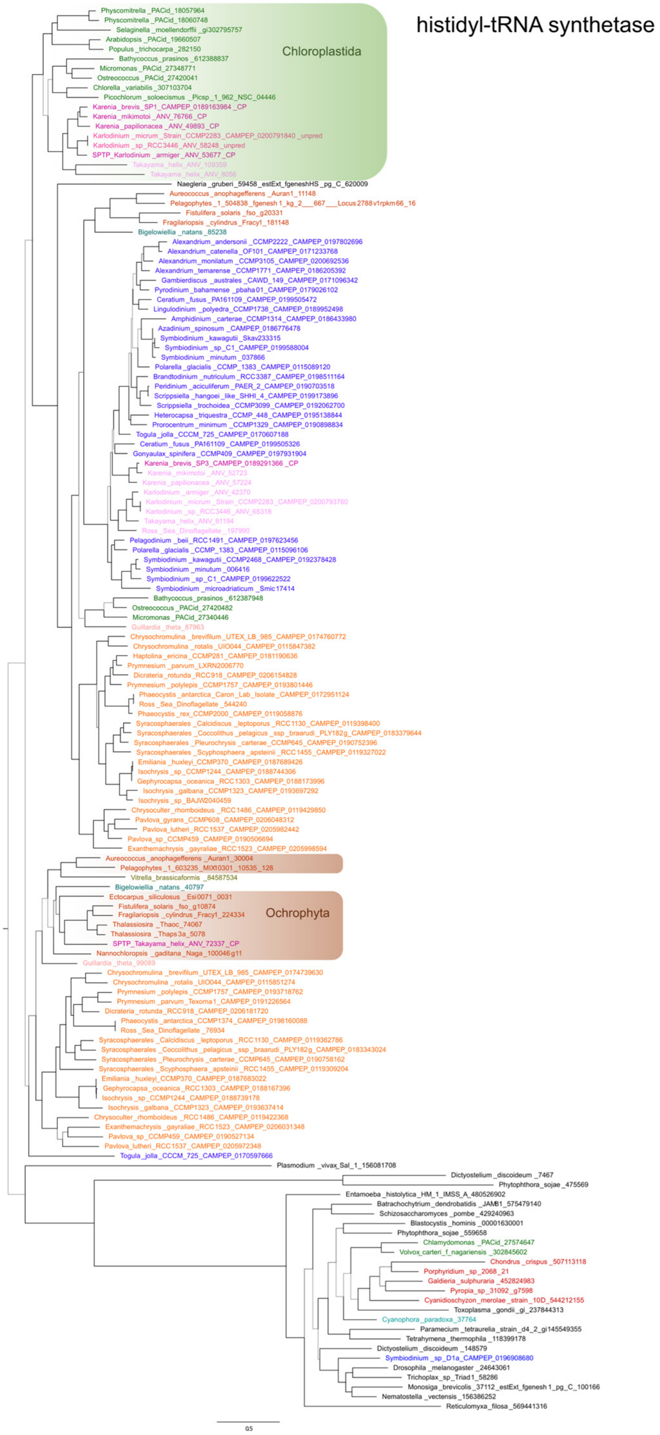

Figure EV4. Automatically generated phylogenetic tree showing the green origin of plastidial histidyl-tRNA synthetases of *Karenia* and *Karlodinium* and brown origin of the plastidial homolog in *Takayama*.

A green-like homolog present in *Takayama* is not predicted as plastidial. The *Karenia brevis* SP3 homolog clustering with the dinoflagellates was likely falsely predicted as plastid-targeted due to an artificial N-terminal extension of the transcript. Bootstrap support is expressed by the branch color (black for ≥90%, dark gray for ≥75%, light gray for <75%). Kareniacean sequences are colored pink with proteins with predicted plastid-targeting signal in darker shades and annotated "CP" suffix; dinoflagellates are coded blue; haptophytes are coded orange; green algae (Chloroplastida) are coded green; and brown algae (Ochrophyta) are coded brown.

 © The Author(s)

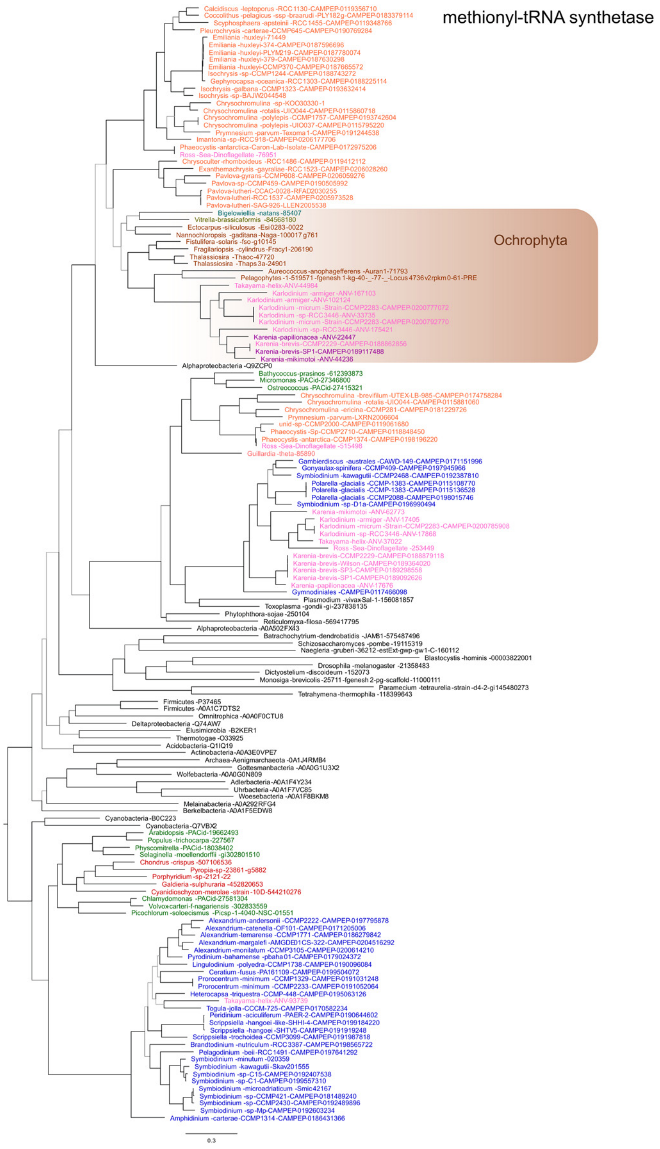

Figure EV5. Automatically generated phylogenetic tree showing the brown origin of plastidial methionyl-tRNA synthetase in all three genera.

Bootstrap support is expressed by the branch color (black for ≥90%, dark gray for ≥75%, and light gray for <75%). Kareniacean sequences are colored pink with proteins with predicted plastid-targeting signal in darker shades and annotated “CP” suffix; dinoflagellates are coded blue; haptophytes are coded orange; and brown algae (Ochrophyta) are coded brown.
